# Early Pars Plana Vitrectomy and Anti-vascular Endothelial Growth Factor (VEGF) in the Management of Terson Syndrome: A Case Linked to Methicillin-Resistant Staphylococcus aureus (MRSA) Meningitis

**DOI:** 10.7759/cureus.94904

**Published:** 2025-10-19

**Authors:** Abdullah Ağın, Dilhan Karaca, Songul Kilic, Feyza Onder

**Affiliations:** 1 Ophthalmology, Haseki Training and Research Hospital, Istanbul, TUR

**Keywords:** anti-vegf, mrsa meningitis, pars plana vitrectomy, sub-ilm hemorrhage, terson syndrome

## Abstract

We present a case of Terson syndrome, characterized by intraocular hemorrhage following acute intracranial events, which poses significant diagnostic and management challenges. The patient was a 45-year-old female with Terson syndrome associated with methicillin-resistant Staphylococcus aureus (MRSA) meningitis and ventriculitis. She presented with vision loss and was found to have sub-internal limiting membrane (sub-ILM) hemorrhages and dense vitreous hemorrhages. Early pars plana vitrectomy (PPV) in the left eye yielded substantial visual improvement. In contrast, delayed intervention in the right eye following anti-vascular endothelial growth factor (VEGF) therapy showed limited recovery. Systemic stabilization was achieved through targeted intravenous antibiotics. This report underscores the critical role of early surgical intervention and multidisciplinary management in optimizing visual and systemic outcomes in complex cases of Terson syndrome. The diagnostic significance of sub-ILM hemorrhage emphasizes its role as a specific marker of severe ocular involvement, necessitating prompt recognition and treatment.

## Introduction

Terson syndrome, initially described by Albert Terson in 1900, is defined by the presence of intraocular hemorrhage associated with acute intracranial bleeding, most commonly subarachnoid hemorrhage (SAH)​ [1]. It results from sudden increases in intracranial pressure, leading to hemorrhages in the vitreous, sub-internal limiting membrane (sub-ILM), or retina [2,3]. The incidence of Terson syndrome following SAH is estimated to range between 8 and 44% [2-4]. The pathophysiology of Terson syndrome is believed to involve abrupt elevations in intracranial pressure, leading to rupture of peripapillary capillaries due to venous congestion and impaired outflow​ [2,3]. Alternative theories, such as glymphatic reflux mechanisms, have also been proposed [2,3]. Clinically, Terson syndrome manifests as sudden visual loss due to vitreous hemorrhage, often accompanied by neurological symptoms secondary to intracranial pathology​ [2,3]. Early diagnosis using ocular ultrasound, optical coherence tomography (OCT), and fluorescein angiography is essential for identifying retinal involvement​ [3].

The management of Terson syndrome involves addressing the underlying intracranial pathology, followed by ophthalmic interventions to restore vision. Conservative approaches may include observation, as spontaneous hemorrhage clearance is possible over several months. However, persistent vitreous hemorrhages frequently necessitate pars plana vitrectomy (PPV) to avert complications such as epiretinal membranes, macular holes, and retinal detachment. Studies have reported significant visual recovery following early vitrectomy, particularly within four to eight weeks of diagnosis​ [2]. We describe a case that illustrates the intricacy of managing Terson syndrome with sub-ILM hemorrhage, particularly in a patient with a history of intracranial aneurysm and methicillin-resistant Staphylococcus aureus (MRSA) meningitis, underscoring the necessity of prompt surgical intervention to enhance visual outcomes. Early PPV led to substantial visual improvement in the left eye. In contrast, delayed intervention in the right eye necessitated anti-vascular endothelial growth factor (VEGF) therapy with plans for future surgery. The multidisciplinary approach combining systemic antibiotic therapy and ophthalmic surgical care highlights the need for individualized treatment strategies to address both ocular and systemic complications.

## Case presentation

**Figure 1:**
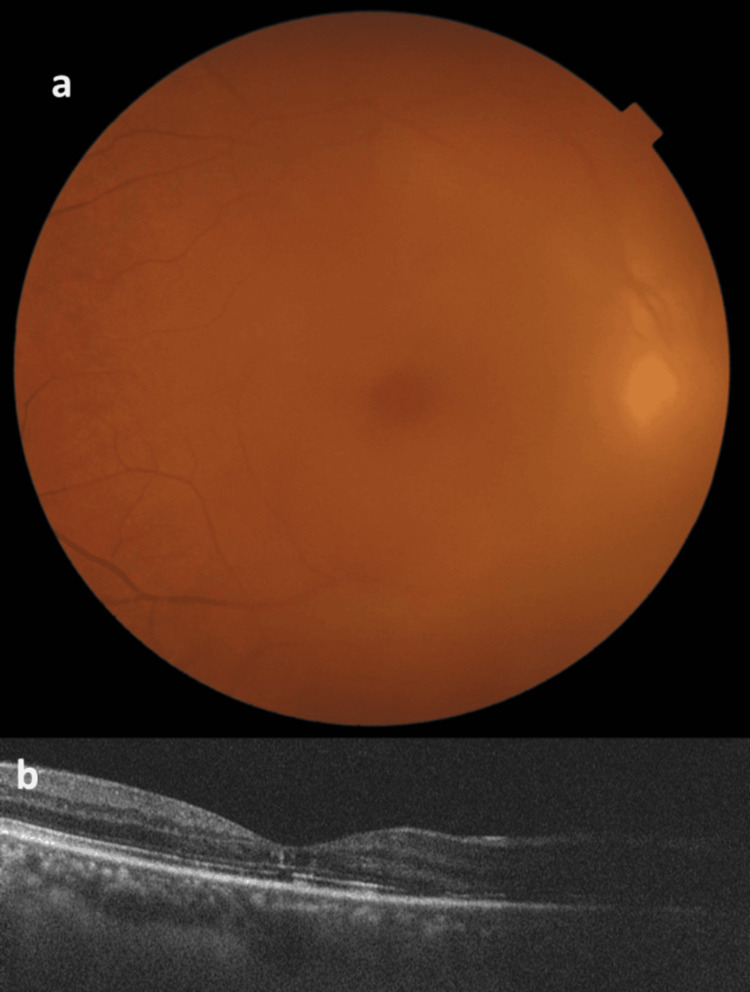
Fundus photograph and OCT scan of the right eye (a) Fundus photograph of the right eye showing vitreous haze consistent with vitreous hemorrhage. (b) OCT scan of the right eye depicting irregularities in the inner retina and disruption in the ellipsoid zone OCT: optical coherence tomography

A 45-year-old female patient presented with complaints of bilateral decreased vision. Ophthalmologic examination revealed that the right eye had a visual acuity of 0.7 logMAR, and the left eye had a visual acuity at the hand-motion level. Anterior segment evaluation showed a transparent cornea and a sclerotic lens in both eyes. Fundus examination revealed vitreous hemorrhage and sub-ILM localized in the peripheral retina; also, posterior hyaloid thickening was noted in the right eye (Figure 1).

**Figure 2:**
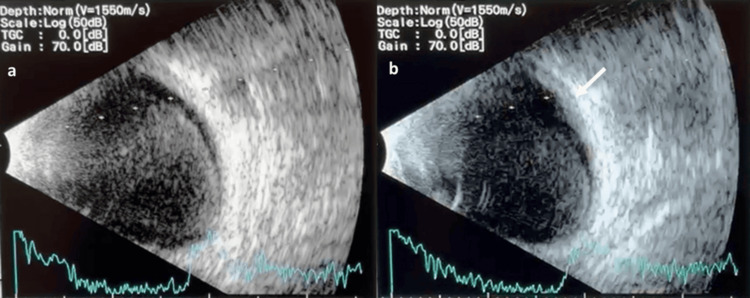
Ultrasound B-scan images of the right eye (a) The image is indicative of vitreous hemorrhage with hyperechoic signals in the vitreous cavity. (b) The image is suggestive of a sub-ILM hemorrhage, as highlighted by the echogenic area (arrow) Sub-ILM: sub-internal limiting membrane

On the other hand, fundus evaluation demonstrated total vitreous haze, which obstructed visualization. Ultrasonography showed hyperechogenicity that confirmed dense vitreous hemorrhage and possible sub-ILM hemorrhage without retinal detachment for the left eye (Figure 2). Systemic evaluation highlighted a history of SAH secondary to a right middle cerebral artery aneurysm, treated with endovascular stenting and coiling. Postoperatively, external ventricular drainage was performed to manage elevated intracranial pressure, which was later removed. However, the patient developed nosocomial MRSA meningitis and ventriculitis. Broad-spectrum intravenous antibiotic therapy was initiated with vancomycin (1 g every 12 hours) and meropenem (2 g every 8 hours). Brain MRI demonstrated ventriculitis and a right frontal abscess.

These findings necessitated continued multidisciplinary management. Laboratory analysis revealed cerebrospinal fluid glucose of 4 mg/dL with serum glucose of 140 mg/dL, elevated protein levels of 1624 mg/dL, and a white cell count of 20,490/mm³, predominantly composed of polymorphonuclear leukocytes. Gram staining showed Gram-positive cocci, and cultures confirmed MRSA. The anti-biotherapy was compatible with MRSA, and the systemic condition improved. Based on the severity of the findings, the left eye underwent PPV under monitored retrobulbar anesthesia after systemic stabilization confirmed by the neurology and infectious disease teams. A standard 25-gauge three-port vitrectomy was performed (Constellation Vision System, Alcon, Fort Worth, TX). Dense vitreous condensation and posterior hyaloid detachment were identified and meticulously removed. Sub-ILM hemorrhages were detected in two focal areas, inferior and superior, to the optic disc around the temporal arcade of vessels. These areas were stained with brilliant blue dye and successfully peeled off using ILM forceps (Finesse Sharkskin ILM forceps, Alcon). A retinal tear was identified at the 11 o’clock position.

**Figure 3:**
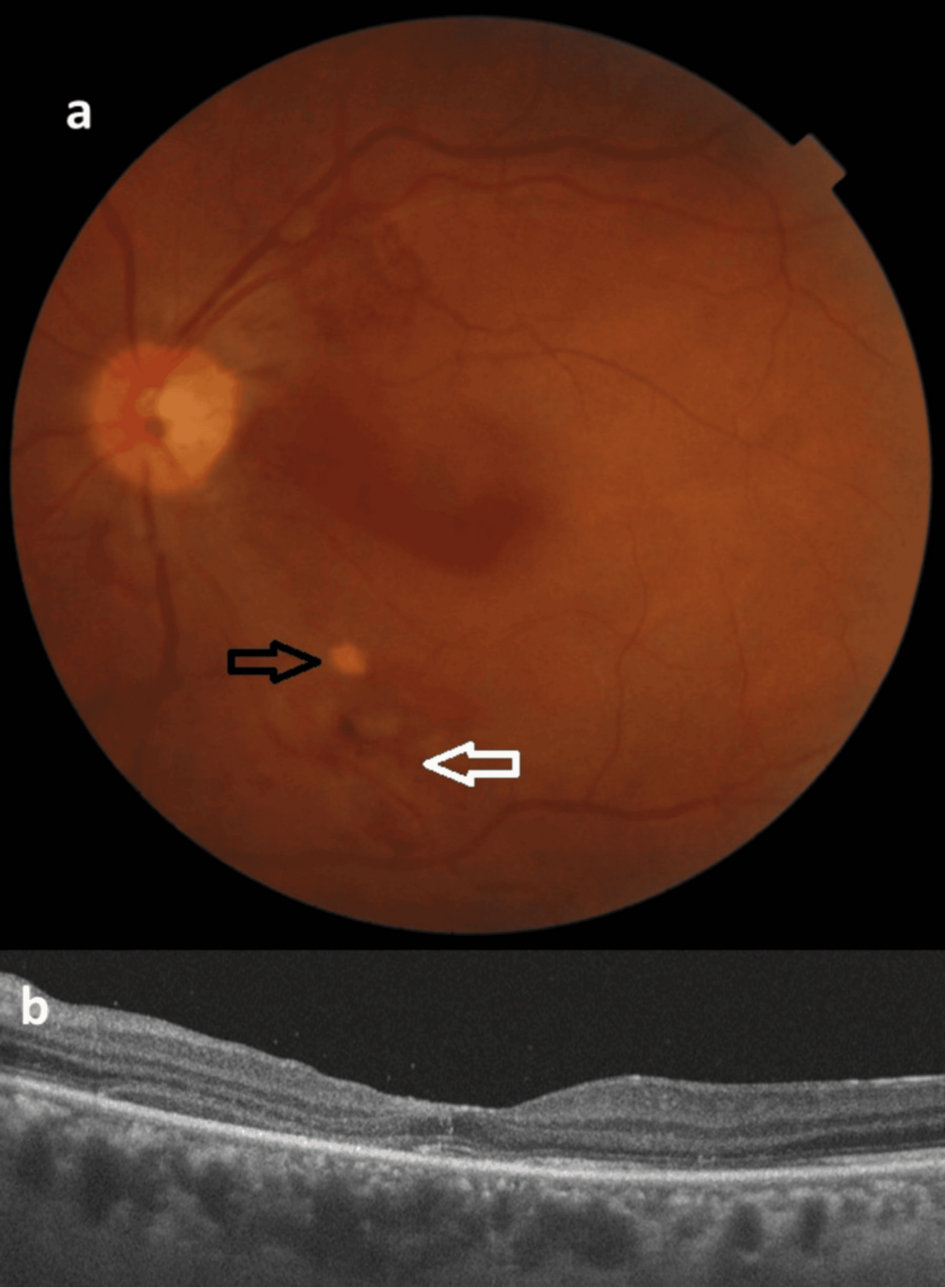
Postoperative fundus photograph and OCT scan of the left eye (a) Postoperative fundus photograph of the left eye showing the inferior and superior temporal arcade regions with appearances related to resolved sub-ILM hemorrhage. Black arrow: residual sub-ILM hemorrhage focus; white arrow: ILM peeled area due to sub-ILM hemorrhage. (b) OCT scan of the left eye demonstrating subfoveal disruption in the ellipsoid zone OCT: optical coherence tomography; sub-ILM: sub-internal limiting membrane

Endolaser photocoagulation for retinopexy was performed in four rows around the retinal tear, with a duration of 0.1 seconds per spot and a power of approximately 200 mW, and fluid-air exchange was completed. The eye was left air-filled without additional gas or silicone oil tamponade. Scleral ports were sutured, and postoperative medications included dexamethasone eye drops eight times a day, tapering off over one month; moxifloxacin eye drops eight times a day for two weeks; and cycloplegic drops twice a day for one month. The left eye showed a significant improvement in visual acuity from hand motion to 0.4 logMAR after one month and 0.15 logMAR after two months. The retina remained attached, and intraocular pressure was within normal limits (Figure 3).

**Figure 4:**
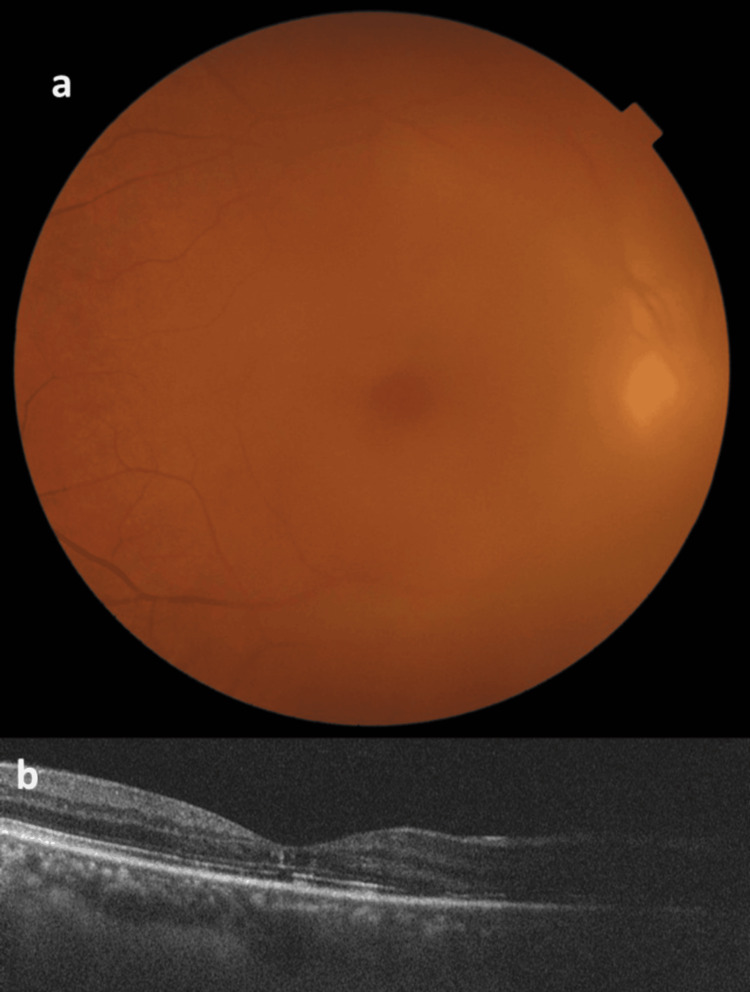
Follow-up fundus photograph and OCT scan of the right eye (a) Fundus photograph of the right eye showing decreased vitreous haze compared to pre-anti-VEGF injection. (b) OCT scan of the right eye depicting irregularities in the inner retina and disruption in the ellipsoid zone. Decreased vitreous haze was noted compared to pre-anti-VEGF injection OCT: optical coherence tomography; VEGF: vascular endothelial growth factor

For the right eye, as the patient reported satisfactory visual function and declined surgical intervention, two intravitreal injections of 1.25 mg/0.05 mL bevacizumab (Altuzan, Roche, Basel, Switzerland) were administered at one-month intervals to reduce vitreous hemorrhage and potential neovascular activity. However, follow-up at two months showed a mild improvement in visual acuity (from 0.4 to 0.3 logMAR), yet visual function remained limited compared to the contralateral eye, with persistent vitreous opacities and posterior hyaloid condensation, suggesting the need for delayed surgical intervention (Figure 4).

Concurrent systemic antibiotic therapy effectively controlled the MRSA meningitis and ventriculitis, stabilizing the patient’s neurological and systemic status. This case highlights the complexity of managing Terson syndrome with distinct treatment approaches and demonstrates the value of timely surgical intervention for optimizing visual outcomes.

## Discussion

This case highlights an unusual and complex presentation involving bilateral vitreous hemorrhage, a rare manifestation requiring a multidisciplinary approach due to concurrent systemic complications. The bilateral presentation in this case contrasts with the usual prevalence, offering an uncommon example. [5]. The co-occurrence of MRSA meningitis further complicated the management, necessitating systemic and ophthalmologic coordination. For instance, one case report has highlighted a similar prevalence, particularly among patients with subarachnoid hemorrhage, reinforcing the need for vigilant assessment and management [6]. The bilateral nature of Terson syndrome makes management more complicated. This feature amplifies the clinical significance of the case, as it underscores the increased severity and need for aggressive management compared to unilateral presentations.

Prevalent etiologies of sub-ILM hemorrhage encompass Valsalva retinopathy, blood dyscrasia, ocular trauma, and rupture of a microaneurysm [7]. Notably, sub-ILM hemorrhage, as observed in this case, was considered a distinct and specific finding in Terson syndrome. Zhang et al. [8] emphasized sub-ILM hemorrhages as indicative of severe ocular involvement. Similarly, Asahi et al. [9] highlighted sub-ILM hemorrhages alongside other retinal findings, reinforcing their diagnostic relevance in such cases. In this case, sub-ILM hemorrhage foci could not be seen biomicroscopically but were only suspected in ultrasonographic evaluation preoperatively due to the intense vitreous hemorrhage in the left eye. Also, intraoperative photography could not be obtained due to the lack of a photography system in the operating room. However, since the peripheral retina was visible in the right eye, there were foci of sub-ILM dehemoglobinized (white) hemorrhage, and a possible chorioretinitis could be suggested in the differential diagnosis for these foci.

Multidisciplinary care was crucial, as systemic complications extended beyond ocular findings. Studies highlight the association between Terson syndrome and poor systemic prognosis due to higher morbidity and mortality rates [2]. Early and accurate diagnosis, followed by individualized treatment, is essential to improving outcomes. The systemic stabilization achieved through targeted antibiotic therapy was vital in this case, illustrating the critical balance between ocular and systemic management.

Regarding ophthalmic management, PPV in the left eye led to significant visual improvement, consistent with studies emphasizing early surgical intervention [5]. PPV has been shown to enhance visual acuity, especially when performed promptly [6]. In contrast, anti-VEGF therapy in the right eye produced limited improvement, reflecting challenges in addressing persistent vitreous opacities and posterior hyaloid condensation. Reports highlight the variable efficacy of anti-VEGF therapy in cases complicated by neovascularization and retinal damage [10]. Conversely, PPV directly resolves vitreous hemorrhage and related retinal abnormalities, often yielding better outcomes. This discrepancy underscores the importance of early surgical intervention over conservative approaches.

This report underscores the importance of identifying ocular manifestations of systemic conditions. Given the high risk of complications, including epiretinal membranes, macular holes, and retinal detachment, early surgical intervention is key to preventing long-term visual impairment [6]. Furthermore, systemic stabilization through appropriate antibiotic therapy was vital, highlighting the interplay between ocular and systemic management.

## Conclusions

This report emphasizes the complexity of Terson syndrome with sub-ILM hemorrhage, particularly in the setting of a bilateral presentation and systemic infections. Contrasting outcomes between the two eyes demonstrate the superiority of early surgical intervention over conservative approaches. Future studies should refine diagnostic tools and therapeutic protocols to optimize outcomes. This report adds to the literature on Terson syndrome, offering insights into its management and reinforcing the need for a multidisciplinary approach.

## References

[1] Terson A (1900). Vitreous hemorrhage and cerebral hemorrhage syndrome (Article in French). Clin Ophthalmol.

[2] McCarron MO, Alberts MJ, McCarron P (2004). A systematic review of Terson's syndrome: frequency and prognosis after subarachnoid haemorrhage. J Neurol Neurosurg Psychiatry.

[3] Regillo CD (1999). Distant trauma with posterior segment effects. Ophthalmology.

[4] Lee GI, Choi KS, Han MH, Byoun HS, Yi HJ, Lee BR (2015). Practical incidence and risk factors of Terson's syndrome: a retrospective analysis in 322 consecutive patients with aneurysmal subarachnoid hemorrhage. J Cerebrovasc Endovasc Neurosurg.

[5] Koman E, Gajda B, Kiszka A, Cisek A, Nowomiejska K, Rejdak R (2016). Effectiveness of vitrectomy in Terson syndrome — case series. Ophthalmol J.

[6] Augsten R, Königsdörffer E, Strobel J (2000). Surgical approach in Terson syndrome: vitreous and retinal findings. Eur J Ophthalmol.

[7] De Maeyer K, Van Ginderdeuren R, Postelmans L, Stalmans P, Van Calster J (2007). Sub-inner limiting membrane haemorrhage: causes and treatment with vitrectomy. Br J Ophthalmol.

[8] Zhang Y, Lei C, Huang X, Zhang M (2024). Delayed macular hole secondary to Terson syndrome: a case report and literature review. J Int Med Res.

[9] Asahi MG, Weiss SJ, Peddada K, Malik D (2019). A case of Terson-like syndrome in a patient with viral meningoencephalitis. Case Rep Ophthalmol Med.

[10] Toffoli D, Allaire GS, Barkat F, Sebag M (2010). A neovascularized epiretinal membrane in a patient with Terson syndrome. Retin Cases Brief Rep.

